# Ethics of Sensor-Based Surveillance of People with Dementia in Clinical Practice

**DOI:** 10.3390/s25072252

**Published:** 2025-04-02

**Authors:** Jacob Lahr, Nicole Schulze, Larissa N. Wüst, Claus Beisbart, Lena C. Bruhin, Marcello Ienca, Tobias Nef, Manuel Trachsel, Stefan Klöppel

**Affiliations:** 1 University Hospital of Old Age Psychiatry and Psychotherapy, University of Bern, 3012 Bern, Switzerland; 2 Institute of Philosophy, University of Bern, 3012 Bern, Switzerland; 3 Center for Artificial Intelligence in Medicine, University of Bern, 3012 Bern, Switzerland; 4 Gerontechnology and Rehabilitation Group, ARTORG Center for Biomedical Engineering Research, University of Bern, 3012 Bern, Switzerland; 5 School of Medicine, Institute for History and Ethics of Medicine, Technical University of Munich, 80333 Munich, Germany; 6 Clinical Ethics Unit, University Hospital Basel (USB) and University Psychiatric Clinics (UPK) Basel, 4031 Basel, Switzerland; 7 Faculty of Medicine, University of Basel, 4056 Basel, Switzerland

**Keywords:** dementia, ethics, sensors, patient surveillance, decision-making capacity, autonomy

## Abstract

Sensor-based surveillance technology (SST) is increasingly employed in the care of people with dementia (PwD) in hospitals, nursing homes, and home care. With improved functionality and availability of SST, there will likely be a growing utilization in clinical settings. In the context of staff shortage, the expected resource-efficient safety benefits are attractive but must be critically balanced against concerns that basic ethical principles are violated. In this discussion paper, we provide a brief overview of frequently used SST and discuss ethical issues associated with their use. We identify intrusion into privacy and the complex interaction between stakeholders as the biggest challenges. Moreover, the frequent lack of decision-making capacity in PwD poses particular ethical challenges. Evidence concerning safety benefits exists mostly for the detection of patients leaving the intended area (i.e., getting up, leaving the premises) or occurring falls. The level of privacy intrusion to detect occurring falls varies widely between different sensor systems. Finally, we propose best practice recommendations for the clinical implementation of SST in the care of PwD, and we address the circumstances under which the use of sensors can be ethically justified despite the absence of legally valid informed consent.

## 1. Introduction

Dementia is characterized by a decline in at least two cognitive domains, which may be memory, executive functioning, attention, language, social cognition and judgment, psychomotor speed, visuoperceptual or visuospatial abilities, or neurobehavioral changes [1]. To address the challenges arising from these cognitive impairments, advances in electronic monitoring technology offer promising solutions to enhance the safety and autonomy of people with dementia (PwD, [2,3]). Technologies such as wrist-worn emergency buttons are already widely used, and relatives of PwD increasingly employ GPS-based tracking devices to prevent wandering incidents of disoriented patients [4], raising several ethical issues [5]. Newly developed contactless ambient sensors, based on radar or laser technology, or pressure-sensitive mattress sensors gradually find their way into hospitals or nursing homes. This trend is accelerating because hospitals and nursing homes are affected by a growing shortage of qualified nursing staff, escalating the already high workload and burden of employees [6,7]. Sensor systems may assist nursing staff in allocating their resources more efficiently.

Surveillance of individual patients using video monitoring might be acceptable for a short time period and under specific circumstances, e.g., in the case of potential self-harm when continuous patient surveillance is required [8]. By contrast, long-term surveillance of patients using video monitoring appears socially and ethically problematic in this context. Yet, there is no consensus for patient surveillance using ambient sensor systems, and the acceptance of patient surveillance in psychiatric units differs between countries and cultures [9]. This absence of a consensus calls for individualized consent. However, with the increasing severity of dementia, the need for monitoring and surveillance rises, while at the same time, the ability to consent decreases. This is true for somatic as well as psychiatric disorders and is best illustrated by extensive monitoring in intensive care units, where no formal consent is acquired but consent is assumed.

A recent unpublished survey including 11 nurses from an old-age psychiatric ward in Switzerland revealed a high consensus on the potential benefits of a sensor-based system to facilitate work, particularly during night shifts, and all participants declared openness to using sensor-based surveillance technology (SST). Responses concerning the most useful information a sensor could provide were far more diverse, with an occurred fall or the intention of a frail patient getting up mentioned most frequently.

So far, ethical considerations are sometimes underrepresented in the development and research on assistive systems for older adults [10,11]. Existing literature often focuses on camera systems for surveillance in nursing homes [12] or home care [13,14,15] or on wearable devices [5]. In clinical research, it is important to include persons who are unable to consent, as they would otherwise be underrepresented in clinical research, resulting in difficulties providing evidence-based medicine for these patients [16]. Nevertheless, including patients who cannot consent in clinical trials raises the challenge of having to assume the patient’s will [16]. Clinical staff also face this challenge when decisions about medical treatments or surveillance measures must be made.

Naturally, ethical aspects of SST in the care of older people must be considered in the development of such technologies, but this topic is beyond the scope of the present paper. For a discussion on ethical principles in the development of SST, we would like to refer to the specific literature [13,17].

However, a lack of attention to ethical aspects can negatively affect the acceptance of such devices [11]. Thus, in this paper, we discuss ethical issues in the context of SST using unobtrusive sensors in the care of PwD. We equally highlight positive and negative aspects. The ethical issues are structured following prior work by Ienca and colleagues [11].

We start by surveying sensor technologies for monitoring PwD in terms of their benefits and degree of privacy intrusion before discussing further relevant ethical issues. We conclude with a proposal for best practices in the clinical use of sensor technology.

## 2. Technologies of Surveillance Systems

Here, we focus on infrared, piezo, radar, and lidar sensors, as these technologies seem to be the most promising for no-touch, unobtrusive ambient surveillance systems.

**Infrared sensors** capture video signals in the infrared wavelength and obtain a thermal image. In such images, humans are clearly recognizable due to their high body temperature. Infrared-based systems have been successfully used in the laboratory [18] and in clinical settings [19] to alarm nursing staff when patients leave their beds. However, as in the case of conventional video surveillance, privacy concerns for monitored individuals are significant, as persons and activities are identifiable without further data processing.

**Pressure-sensitive piezo sensors** can be placed underneath the patient’s mattress and allow for distinguishing whether a patient is lying or sitting in bed and further to detect movements. Additionally, sleep stages can be determined from piezo sensors under the mattress, though these are less accurate than polysomnography, the gold standard of sleep analysis [20]. Still, the data quality from piezoelectric sensors, similar to that of wearable sensors, is comparable to, or even better than, actigraphy, which is currently the clinical standard for mobile sleep assessment [21]. On a geriatric ward, an alarm system using piezo sensors performed well in detecting when patients tried to leave their beds but still showed a substantial amount of false alarms for bed exit attempts (32%, [22]). Nevertheless, the nursing staff perceived the system positively, and the pressure sensor is arguably a far less invasive method than a camera system.

**Radar sensors** use high-frequency electromagnetic waves to detect the position and movements of individuals and objects. In laboratory studies, radar sensors have been shown to be able to detect falls with high sensitivity and specificity, i.e., fall events were reliably detected with only a few false alarms [23,24]. In nursing homes, radar systems can detect falls but still show a rather large number of false alarms (above 50%, [25]). Fall alerts allow prompt responses in nursing homes and hospitals and thus potentially prevent further harm to the patients, but false alarms might mitigate the acceptance of the devices. Among non-obtrusive sensor techniques, radar may have benefits as it covers broad areas and is easier to install than pressure sensors on the floor. In Switzerland, radar-based sensors are widely used to detect out-of-bed events and falls in nursing homes (Qumea, Qumea AG, Solothurn, Switzerland).

**Lidar sensors** use rotating laser pulses to create a two-dimensional image of the surroundings and can be used to detect various activities, such as fall events [26], or even to analyze gait parameters [27].

Regarding privacy concerns, radar and lidar systems have the advantage that they do not create a direct image from which it would be possible to identify individual persons recorded. Nevertheless, all the above-mentioned technologies require the individual who is to be surveilled to be in the range of the sensor, which might be compromised by objects placed between the person and the sensor.

Although unobtrusive sensor technology is widely used in nursing homes and clinics, few studies have thoroughly examined its application in a clinical setting [28]. To our knowledge, existing research on ambient sensors only demonstrates the feasibility of the technology without evaluating it with specific patient populations, such as PwD, and lacks patient-related outcome measures or the sample size, and thus the number of fall incidents is very small, limiting its statistical power [25]. Further research on the efficacy of sensor-based surveillance is still ongoing, and preliminary results indicate that the number of falls can be reduced by 50% [29].

Falls and fall-related injuries are a major concern in healthcare for older people [30,31]. In an acute hospital setting, nurses report that it would be nearly impossible to prevent PwD from falling without the patients being under continuous one-to-one surveillance [32]. Continuous surveillance through clinical staff would not only entail immense personnel costs but would also be a substantial intrusion into a patient’s privacy. The use of unobtrusive sensor systems that do not allow the identification of individuals might provide a good compromise to enhance the safety of PwD while maintaining privacy and autonomy as far as possible. Nevertheless, it must be noted that sensors will not be able to prevent all falls that continuous surveillance through nurses might be able to.

## 3. Ethical Issues and Principles

A review investigating which ethical aspects were considered in studies reporting on intelligent assistive living technologies for PwD found six thematic groups addressed in the order of declining importance: **autonomy, non-maleficence, beneficence, justice, interdependence, and privacy,** to which we add **avoidance of stigma** [11].

Autonomy, non-maleficence, beneficence, and justice are the four principles of biomedical ethics [33]. Subordinate to these fundamental ethical principles, interdependence, privacy, and avoidance of stigma pose specific ethical challenges when using SST in the care of PwD, for which reason we discuss them separately.

**Autonomy** is concerned with the idea of self-government [34], i.e., that a person is in charge of their behavior and actions without being under the control of others. **Non-maleficence** refers to the “primum nihil nocere” and a moral obligation to avoid or, at least, minimize the causing of harm. **Beneficence** focuses on the promotion of benefits and welfare in the person, while **justice** requires a fair distribution of risks, costs, and benefits among the involved parties [11], which in our case would be the patients, their relatives, medical professionals, and also the providers of surveillance technology.

**Interdependence** means the integration in a group of people who share similar goals and principles and who depend on each other while keeping some independence [35]. **Privacy** is the power to decide what information about oneself and one’s activities is disseminated to others [11]. Lastly, we have added **avoidance of stigma** [10] as SST can equally contribute to or reduce stigma.

The extent to which these principles and issues are relevant depends on the concrete application of SST and the context (e.g., hospital, nursing home, or home care). As will be shown below, the same technology may well be justifiable or ethically problematic depending on the extent of data acquisition, anonymization, and storage.

### 3.1. Specific Ethical Issues in Patient Surveillance

Specific ethical issues arising in patient surveillance are discussed in the following section. A summary is presented in Table 1.

#### 3.1.1. Autonomy

In contrast to, e.g., assistive robots, SST does not have a direct impact on patient autonomy because its primary role is to aid others in the care. Still, there can be an indirect impact that is mediated via the actions of caregivers or healthcare professionals. When a surveillance system has informed caregivers that PwD show a certain behavior, the caregivers may support the PwD in the realization of their aims, or they may interfere with them to stop what they are doing. Accordingly, SSTs can indirectly increase or diminish patient autonomy. They are particularly supportive of autonomy if they replace measures that restrict PwD autonomy more strongly (e.g., replacing physical restraint with sensors to prevent falls). All this at least holds if autonomy means that one’s actions are not interfered with. We realize that decisions of PwD can generally be questioned concerning autonomy based upon a lack of decision-making capacity, but discussing this aspect is beyond the scope of this paper. SSTs do not count as measures restricting freedom of movement (e.g., according to Art. 438 of the Swiss Civil Code) themselves, but the restriction that may be a consequence of their use can be, underlining the need for clear guidelines to regulate SST use. Therefore, we state recommendations on how to use SST to support patient autonomy below.

#### 3.1.2. Non-Maleficence

SSTs do not harm people directly. Some technologies, e.g., infrared imaging, do not interfere with the patient at all; for other technologies, e.g., lidars and their laser pulses, there is no evidence of harm. Note, too, that many surveillance technologies are not at all or only barely noticeable to the patient, though disturbance through the mere presence of devices cannot be expected. Only if the information obtained via technology is used to harm a person is non-maleficence violated. However, in hospitals and nursing homes, information is presumably not used in a harmful way; at most, based on the information from surveillance techniques, healthcare professionals may take measures that are harmful in the short term to prevent more severe or longer-lasting harm. We thus conclude that non-maleficence is not an issue for SST in clinical practice.

#### 3.1.3. Beneficence

While the mere presence of sensors does not improve a patient’s quality of life, sensors can contribute to enhancing the quality of life if their information is used appropriately. In this way, patient safety is increased by preventing patients from negative events and risks thereof (as defined by reference [8]). Such events may be falls, getting lost, or, more generally, a worse health outcome. Furthermore, the allocation of available caregiving personnel could be optimized, benefiting all patients. Even though sensor systems for patient surveillance are increasingly used in hospitals and nursing homes, scientific evidence of the benefits remains sparse. Using fall detection sensors allowed an immediate response to a fall of a patient, compared to delayed fall recognition and medical assistance in unsupervised patients falling in a control group without sensor surveillance [25].

Sensor surveillance systems also benefit caregivers. Their fear that their patients may have an accident and their sense of responsibility is a constant burden. Sensor surveillance provides relief for related sorrows.

#### 3.1.4. Justice

The principle of justice concerns the fair distribution of healthcare resources. Current sensor surveillance solutions are still rather expensive; the costs per year can amount to more than USD 1000, potentially creating disparities in access. High costs may limit the availability of SST to only those who can afford them or those in well-funded institutions, raising ethical concerns about equality in care [36]. Accordingly, the technology will not be available to every patient in in-home care. In nursing homes, the cost of implementing these systems could significantly impact budgets, possibly diverting funds from other essential services. This economic burden may lead to unequal access among patients, with wealthier individuals receiving better surveillance and, consequently, better care and higher safety. This presents an even bigger issue in low- to middle-income countries [37], where technologies might be unaffordable due to their high costs. Addressing these disparities requires considering ways to make these technologies more affordable and accessible to all patients, regardless of their financial status. For a detailed discussion of this principle, we refer to other literature (e.g., reference [36]), as this is beyond the scope of the present paper.

In situations where not all patients can be given access to SST, it is necessary to determine which patients will benefit from being given access to SST. To date, there is not even a consensus on how to determine the benefits of SST for any specific individual. The reason is a lack of systematic research on the application of SST for broad populations of patients; thus, there are no relevant recommendations in national guidelines.

#### 3.1.5. Interdependence

Interdependence refers to the valuable mutual relations between PwD and their caregivers or healthcare providers. While PwD frequently rely on assistance to fulfill their daily needs, this reliance does not preclude encouraging PwD towards independence whenever possible.

SST can have various effects on the network of relations between the people affected and, thus, on interdependence. On the positive side, caregivers and family members are exonerated from the permanent duty to survey PwD. This leaves them with more resources to build and maintain valuable relationships with those directly affected. Especially in ambient living environments, SST can provide peace of mind to family members by ensuring that emergency detection is in place. Patients are also willing to use surveillance technology and accept intrusions of their privacy if these technologies reduce the burden for family carers [38]. On the negative side, during a day shift, a system that initiates patient-staff interactions only based on an alarm may reduce social interactions between caregivers and patients. Another problem is that caregivers and family members obtain more data from the patients, which may make the latter more dependent on the former.

When it comes to human relationships, we must not neglect that new stakeholders become involved, namely the manufacturers and sellers. Besides wanting to sell their products, manufacturers might be interested in collecting as much data as possible from their devices to improve their devices and algorithms, develop new products, or optimize sales strategies. The high number of stakeholders may also lead to a diffusion of responsibilities. The question, ethically and legally, arises as to what extent the assistive devices or their manufacturer can be held responsible in case of malfunctions or non-detection of adverse events. Usually, manufacturers will put in place terms of use, transferring responsibility to the users as far as possible [39].

#### 3.1.6. Privacy

Privacy is a significant ethical concern with SST. Any surveillance of a patient interferes with the patient’s privacy when personal data are acquired, stored, and further processed. The level of intrusion varies with the type of technology and the data collected. Data coverage ranges from, e.g., 24/7 video monitoring to automated heart rate measurements once a day. The extent of privacy intrusion also depends on the extent to which data are disseminated: the collection of video data is far less problematic if it serves only to detect a fallen individual by an AI in real-time than a system transferring data to TVs in the nurses’ room where visitors might see it. Here, video streaming will typically allow the identification of patients, involved staff members, and visitors, which could not be the case for radar or lidar unless substantial efforts are undertaken. A special group of people to whom data might be made available are relatives of PwD. The medico-legal system in Switzerland gives relatives the right to decide for an incapacitated patient, and this role legitimizes access to certain sensor data. While it can typically be assumed that relatives and caregivers prioritize the patient’s well-being, their curiosity concerning the PwD’s behavior may additionally motivate the quest for information that should be kept private.

The severity of privacy violation further depends on the storage of collected data. Options range from no storage (i.e., real-time analysis only) to an unclearly regulated cloud server hosted abroad. The initiator of the surveillance measures can usually be assumed to have access to collected data, but service providers may also obtain access to these data. Surveilled patients may not know what data are collected and who can access the data. The same can be true for relatives who agree to sensor-based monitoring. While the patient keeps a certain level of control over data collection when using wearable devices (e.g., by not always wearing the device), the patient’s control over data collection through ambient sensors is limited to giving or not giving consent or, when possible, staying in areas that are not captured by sensors. Additionally, the low visibility of sensors makes it more likely that the surveilled individuals forget about their presence. In consequence, surveillance systems might, intentionally or not, provide the relatives of the PwD information and insights into the patient’s life, which the patient would prefer not to share.

Of course, the acquisition and storage of data only pose a threat to privacy if it is possible to identify individuals in the sensor data. Therefore, SST that does not allow identifying a person should be preferred over SST that allows identification with little effort. For example, radar sensors with rather abstract data recorded might be preferred over video-based surveillance systems.

Overall, the amount of data collected about PwD through ambient sensors raises legitimate worries about privacy, and the measurement and storage of the data seem only permitted if valid informed consent is given. In other words, installed technology must be deactivated if a competent patient withholds consent. However, people with advanced dementia are typically incapable of deciding whether or not to use SST. This results in medical personnel and, if available, relatives or guardians of the patients being required to make decisions based on the presumed or documented patient’s will.

Of course, sensors affect not only the privacy of PwD but also that of caregivers when data of caregivers are recorded, possibly even without them knowing. This issue may become increasingly relevant when more sophisticated sensors can help document the actions of healthcare professionals. This may support the documentation of the care workers’ work, but a detailed documentation of time spent in the recreation room warrants caution. Different legal issues arise if the sensors document mistakes made by caregivers. Documentation of nursing measures through sensors could also be used to detect errors made by healthcare and protect patients from abuse. At the same time, healthcare professionals could be protected from unwarranted accusations of misbehavior toward patients [36].

#### 3.1.7. Avoidance of Stigma

Stigma refers to a negative attitude based on prejudice and misinformation that is directed toward individuals, e.g., those with mental health conditions. It is triggered by a marker of illness (such as odd behavior or signs of treatment) and results in social exclusion and reduced access to care [40].

Therefore, stigmatization is a concern with any visible monitoring system. SSTs that are unobtrusive, such as ambient sensors, are less likely to cause stigma compared to more noticeable devices like pressure-sensitive mats placed in front of beds. Reducing the visibility of these technologies can help minimize the social stigma associated with being under constant surveillance, thereby maintaining the dignity of PwD.

However, the balance between unobtrusiveness and effectiveness is crucial. While less noticeable sensors are preferable, they must still provide accurate and reliable data to ensure patient safety. The design and implementation of these systems should aim to minimize visibility while maximizing their functional benefits to avoid any stigmatization. Furthermore, it must be assured that visitors are informed if they might be identified from recorded data.

## 4. Best Practice Recommendations

Following the discussion above, we identify beneficence, privacy, autonomy, and interdependence as the issues most affected when using SST. These factors pull in different directions: while SST indirectly contributes to patient welfare and benefits caregivers, too, there are costs in privacy, and the impact on autonomy and interdependence can be double-edged. Accordingly, tradeoffs between the principles are necessary. Given the inability of most PwD to autonomously decide for themselves, decisions on the tradeoffs cannot be left to the patient.

In developing our recommendations, we are building upon previously proposed guidelines for the use of assistive technology [41] and expanding and specifying them to the use of unobtrusive SST in PwD. Thereby, we aim to provide recommendations that will simplify the decision for the use of SST in individual cases. A significant hurdle to a thorough evaluation of these tradeoffs for every individual case is the limited ability of nursing homes and hospitals to conduct highly individualized ethical evaluations, given the limited resources and the need to standardize processes. For the same reasons, these facilities will not be able to forgo the use of sensor technology.

In their proposed principles for the use of surveillance technology, Fisk puts an emphasis on the use of cameras for surveillance [12], which, in our view, is a much greater intrusion into individual privacy due to the potential to identify individuals compared to the SST we are focusing on. These privacy intrusion issues do not only concern the PwD, but also staff and visitors.

Therefore, in what follows, we propose concrete and feasible best practice recommendations for the application of unobtrusive SST in care facilities for older people:

(1) **Patient well-being**: Employing sensor systems only if the probability of patient benefit is high. Here, indirect benefits are crucial; e.g., when sensor data helps to support a more effective use of time for the team of healthcare professionals, patients will benefit indirectly.
(2) **Data minimization**: Only storing data that is necessary for ensuring the patient’s best interest and consider setting up a system with automatic data purging, which removes data after a predefined period. Concerns about the violation of privacy can also partially be avoided by (pseudo-)anonymizing data, as, according to, e.g., European (recital 26, General Data Protection Regulation) and Swiss law (Art. 2, Human Research Act), anonymized data are no longer considered personal data and, therefore, are not subject to the requirement of data minimization. Furthermore, if several systems with comparable performance are available, the system that assesses the least amount of data and is least obtrusive should be chosen.
(3) **Data security:** Using up-to-date storage systems with restricted access rights and security measures such as two-factor authorization where applicable. Considering the trade-off between local and cloud-based data analysis and storage, critically balance potential security flaws against advantages in usability and manageability. Unauthorized identification of patients from sensor data must be prevented.
(4) **Open design:** Developing hardware and software in adherence to the criteria of open design. This may contribute to data minimization and data security but often stands against the financial interests of manufacturers and service providers.
(5) **Transparency vs. stigma:** Informing patients, caregivers, and relatives if they are being recorded by sensors. At the same time, avoid stigma by making the surveillance unknown to people who are not recorded or involved in the treatment of the patient (i.e., avoid signs on the outside of the room door that indicate the use of SST).
(6) **Autonomy:** Involving relatives as indicators of the presumed patient’s will in decisions that the patient is no longer able to make due to decision-making incapacity. Strive to consider the patient’s values based on expressed wishes and principles to warrant a patient-centered approach to care. While the awareness of health data privacy concerns may be low in generations that did not grow up using the internet daily, the assumption that sensor data should be analyzed and stored for the maintenance of health appears justifiable, especially since no significant harm to the individuals involved is evident. Formally, in the psychiatric hospital setting, the usage of SST should be mentioned in the treatment plan. Although we do not consider SST as such a direct restriction of movement, they may be used to restrict personal freedom.
(7) **Social consensus**: Discussing surveillance measures with all stakeholders and trying to obtain consensus, which varies depending on cultural aspects and evolves over time. Concerning health data privacy, not only the legislature but also patient concerns about health data privacy vary significantly between countries and continents [9].

### Implications for Clinical Practice

Many inpatients in old age psychiatry suffer from advanced stages of dementia and are at high risk of experiencing falls. This risk is elevated by the acute medical condition and probably the unfamiliar environment. Given the inability to notice their high risk of falling paired with an inability to provide consent, we recommend that an unobtrusive sensor be used for such a patient, alerting the nursing staff when the patient tries to exit their bed. By arriving in the patient’s room while the patient is exiting the bed, the nursing staff can assist the patient if needed and therefore prevent the patient from falling. This can be assumed to be in the patient’s best interest. Where feasible, relatives should be informed, and visitors should be made aware of the continuous monitoring.

The Swiss guidelines state that a person-centered and caring position of clinical staff is a key factor in the care for PwD [42]. Therefore, thorough education of clinical staff is necessary so that SST will be used to improve care and enhance individual autonomy by providing help and protecting individuals from falling. Clinical staff plays a crucial role in supporting patient autonomy [43], which is not only true for nursing homes but also in hospitals or for home care. It will be important that alarms triggered by SST support patients in their intended actions and thus enhance autonomy, instead of forcing patients to return to their bed after an alarm is raised. Furthermore, technologies need to be designed to minimize the number of false alarms to prevent an unnecessary increase in workload and caregiver burden and alarm fatigue [44]. Nursing staff needs to be educated in handling false alarms and should be given the chance to provide feedback to decision-makers and developers. Evaluations of false alarm rates should be performed regularly in the clinical application of SST.

When the health status of a patient changes, the benefit for the patient from SST can vary. Therefore, it is important that the need for the use and the extent of used surveillance technology be reevaluated regularly.

There is currently no clear consensus on whether certain types of SST can be considered a clinical standard, as vital sign monitoring in hospitals already is. We argue here that using unobtrusive sensor technology in the care for PwD, especially when they have an increased risk of falling, should be established as a clinical standard. Unobtrusive sensor technology can help to reduce the number of falls and thus fall-related injuries and support a timely provision of medical care in the event of a fall, while intrusion to the individual’s privacy and autonomy is kept as minimal as possible. Importantly, it might be easier to establish such technology in hospitals and nursing homes, as the personal living space of PwD in the institutional setting is smaller compared to homes, and nursing staff is available immediately to respond to potential alarms raised through the technology. In home use, unobtrusive sensor technology could be equally beneficial, but the implementation might be more difficult due to multiple rooms that possibly need to be covered by sensors and remote persons who would be alerted.

While SST with unobtrusive sensors appears to be an appealing and convenient solution in hospitals and nursing homes due to limited personal space (usually one bedroom), its use in home care may be more challenging. Home environments often require the coverage of multiple rooms, necessitating more expensive multi-sensor systems. Therefore, developing cost-effective sensor solutions for home care is essential. Furthermore, it is important to be transparent about what data are assessed through the sensors, how and how long obtained data are stored, and who has access to the data.

Finally, the abovementioned ethical concerns must be carefully weighed against the potential benefits of SST.

## 5. Conclusions

The integration of SST in the care of PwD presents both promising benefits and important ethical challenges. As healthcare systems increasingly adopt these technologies, it is essential to balance beneficence, privacy, and autonomy.

Sensor-based systems offer substantial benefits, enhancing patient safety by enabling timely interventions and reducing the risks of falls and wandering. They also support caregivers by alleviating constant vigilance and allowing a more efficient allocation of resources, ultimately contributing to a higher quality of life for both patients and caregivers. However, these advantages come at the cost of patient privacy, particularly when installed in long-term care, and can complicate the web of interdependencies among patients, caregivers, family members, and technology manufacturers.

Autonomy is indirectly impacted as caregivers make decisions based on surveillance data, potentially supporting or restricting the patient’s actions. Non-maleficence is generally upheld, as the surveillance systems themselves do not cause harm; however, the misuse of information can lead to restrictive measures that might distress patients. Justice is a concern due to the high costs of these technologies, which may limit access and exacerbate disparities in care. To navigate these ethical complexities, we proposed seven best practice recommendations to ensure that the deployment of sensor-based surveillance in the care of PwD is ethical, effective, and aligned with the best interests of all parties involved. Balancing these considerations will help harness the benefits of technology while mitigating its ethical pitfalls, ultimately leading to more compassionate and equitable care for PwD. Comprehensive staff education is mandatory for the correct implementation of SST in a clinical context and adherence to ethical principles.

## Figures and Tables

**Table 1:** Support and violations of ethical issues in sensor-based surveillance technology.

Ethical Issue	Support	Violation
Autonomy	Support of caregivers to provide timely assistance	Response of caregivers by applying restraints
Non-maleficence	No direct harm from SST	SST is used to harm individuals
Beneficence	Increase in quality of life through improvement of care	Scientific evidence for the benefit of SST in dementia is sparse
	Reduction in caregiver burden	
Justice	Provision of SST based on need rather than financial resources	Socioeconomic inequality affects access to SST
Interdependence	Potential to use resources to build meaningful relationships instead of surveillance	Potential of reduction in human interaction
		Dependence on stakeholders with potential conflicts of interest (e.g., financial interests of manufacturers)
Privacy	Better protection by use of SST compared to human surveillance or cameras	Unauthorized identification of individuals must be prevented
		Data from additional persons (e.g., caregivers, visitors) can also be recorded
		The degree of violation depends on technology and data storage
Avoidance of stigma	Unobtrusive SST can avoid stigma	Visible SST may stigmatize PwD

SST: sensor-based surveillance technology; PwD: people with dementia.

## Abbreviations

The following abbreviations are used in this manuscript:

PwD	people with dementia
SST	sensor-based surveillance technology
LiDAR	light detection and ranging
